# The roots of future rice harvests

**DOI:** 10.1186/s12284-014-0029-y

**Published:** 2014-12-10

**Authors:** Nourollah Ahmadi, Alain Audebert, Malcolm J Bennett, Anthony Bishopp, Antonio Costa de Oliveira, Brigitte Courtois, Abdala Diedhiou, Anne Diévart, Pascal Gantet, Alain Ghesquière, Emmanuel Guiderdoni, Amelia Henry, Yoshiaki Inukai, Leon Kochian, Laurent Laplaze, Mikael Lucas, Doan Trung Luu, Baboucarr Manneh, Xiaorong Mo, Raveendran Muthurajan, Christophe Périn, Adam Price, Sabariappan Robin, Hervé Sentenac, Bassirou Sine, Yusaku Uga, Anne Aliénor Véry, Matthias Wissuwa, Ping Wu, Jian Xu

**Affiliations:** CIRAD, UMR AGAP, Montpellier Cedex 5, 34398 France; Centre for Plant Integrative Biology, University of Nottingham, Loughborough, LE12 5RD UK; UFPel, Plant Genomics and Breeding Center, Pelotas, Brazil; Université Cheikh Anta Diop (UCAD), Département de Biologie Végétale, Laboratoire Commun de Microbiologie IRD/ISRA/UCAD, Centre de Recherche de Bel Air - BP 1386, CP 18524 Dakar, Sénégal; Laboratoire Mixte International Adaptation des Plantes et microorganismes associés aux Stress Environnementaux (LAPSE), Centre de Recherche de Bel Air - BP 1386, CP 18524 Dakar, Sénégal; Université Montpellier 2, UMR DIADE, Montpellier, France; IRD, LMI RICE, USTH, Agronomical Genetics Institute, Hanoi, Vietnam; IRD, UMR DIADE, Montpellier, France; IRRI, Los Baños, Philippines; International Cooperation Center for Agricultural Education (ICCAE), Nagoya University, Furo-cho, Chikusa 464-8601 Nagoya, Japan; Robert W. Holley Center for Agriculture and Health, USDA-ARS and Department of Plant Biology, Cornell University, Ithaca, 14853 NY USA; Biochimie et Physiologie Moléculaire des Plantes, Institut de Biologie Intégrative des Plantes, UMR 5004 CNRS/386 INRA/Montpellier SupAgro/Université Montpellier 2, F-34060 Montpellier Cedex 2, France; Africa Rice Center, AfricaRice Sahel Regional Station, B.P. 96, St Louis, Senegal; State Key Laboratory of Plant Physiology and Biochemistry, College of Life Science, Zhejiang University, Hangzhou, 310058 China; Tamil Nadu Agricultural University, Coimbatore, 641003 India; University of Aberdeen, Aberdeen, AB24 3UU UK; ISRA, CERAAS, Thiès, Senegal; National Institute of Agrobiological Sciences (NIAS), 2-1-2 Kannondai, Tsukuba, 305-8602 Ibaraki, Japan; Japan International Research Center for Agricultural Sciences (JIRCAS), 1-1 Ohwashi, Tsukuba, 305-8686 Japan; Department of Biological Sciences and NUS Centre for BioImaging Sciences, Faculty of Science, National University of Singapore, Singapore 117543 Singapore

**Keywords:** Ideotype, Breeding, Phenotyping, Genetic and molecular controls, Rice, Roots

## Abstract

Rice production faces the challenge to be enhanced by 50% by year 2030 to meet the growth of the population in rice-eating countries. Whereas yield of cereal crops tend to reach plateaus and a yield is likely to be deeply affected by climate instability and resource scarcity in the coming decades, building rice cultivars harboring root systems that can maintain performance by capturing water and nutrient resources unevenly distributed is a major breeding target. Taking advantage of gathering a community of rice root biologists in a Global Rice Science Partnership workshop held in Montpellier, France, we present here the recent progresses accomplished in this area and focal points where an international network of laboratories should direct their efforts.

## Review

The root system has the crucial role of extracting nutrients and water through a complex interplay with soil biogeochemical properties, and of maintaining these functions under a wide range of stress scenarios to ensure plant survival and reproduction. This role is made even more important due to increasing climate instability and limitation of fertilizers and irrigation in cropping systems. Determining the precise contribution of root traits to final grain yield under these scenarios and breeding cultivars harboring root systems adapted to stress profiles prevalent in representative target soil environments are therefore a priority in the plant breeding research agenda. Long neglected, the biology and ecology of roots - the “hidden half” - have recently attracted an increasing number of research groups and disciplines ranging from genetics, molecular biology, cell biology, physiology, microbiology, engineering, and biomathematics. This attraction has been exemplified by the high attendance of the International Root Research Society meetings (http://www.rootresearch.org). Demonstrations at the field level that root anatomical and architectural traits can considerably enhance cereal crop yield stability under limited water and nutrient resources (Lopes and Reynolds [2010], York et al. [2013], Lynch et al. [2014]), as well as the development of high-throughput root phenotyping systems (Clark et al. [2013], Topp et al. [2013]) have also recently contributed to this interest. Furthermore, more groups working in root development in the model plant *Arabidopsis* are now turning to crops, such as rice (*Oryza sativa* L.), the model cereal.

Addressing issues of water and nutrient limitations in rice, the staple food for more than half of mankind, is of particular relevance since rice is cultivated under the widest range of agro-systems among cereals - from irrigated anaerobic conditions to upland aerobic conditions, including flood-prone and drought-prone environments. Breeding for high-yielding irrigated rice has favored shallow root system phenotypes that easily capture resources in the topsoil layer whereas breeding for low input upland conditions has, conversely, favored deep root systems with strong foraging capacity and nutrient extraction ability from poor soil layers. In addition to these divergent root architectures that have been selected for among rice agro-ecosystems, the increasingly fluctuating levels of stress that is projected to occur across rice growing systems may necessitate more diverse root architectures and better adaptability to uneven distribution of soil resources. The diversity of rice for adaptation to different water regimes and soil conditions has favored the identification of genes and quantitative trait loci (QTLs) underlying root development and resource uptake traits. In parallel, methodological progress in the development of experimental set ups that may mimic uneven resource distribution, has led to the establishment of diverse and complementary phenotyping platforms for screening for root traits in diversity panels, mapping populations and mutant resources. Though their relevance to field soil conditions has to be considered with caution, such platforms are instrumental for feeding, then calibrating and validating, dynamic models of root architecture in response to resource scarcity and distribution (reviewed in Hill et al. [2013]). Modeling is likely to be of invaluable assistance for breeding ideotypes adapted to specific soil environments and stress scenarios as millions of simulations can be run encompassing a wide range of environmental conditions (Postma et al. [2014]).

To review the progress in these areas and delineate future priorities where research efforts on rice roots should focus, a workshop gathering 40 participants was organized in the Agropolis campus, Montpellier France, from May 15-16th 2014, in the framework of the Global Rice Science Partnership (GRiSP). The group has first focused its debates on advances in root development and ideotype breeding. As a major output of the discussions, we recommend research areas on which emphasis should be placed that constitute a priority set of core actions of a rice root research collaborative network.

### Accelerate the discovery of genes underlying root structural and functional traits

During the past two years, the rice research community has seen major breakthroughs in the identification of genes behind root response to stress; *DRO1*, a root depth QTL (Uga et al., [2013]) and *PUP1* a root QTL for phosphorus uptake (Gamuyao et al., [2012]) were cloned, and fine mapping of several other rice root/stress response QTLs is ongoing (*DRO2*, *DRO3*, *CHR9*; Y. Uga and N. Ahmadi et al. unpublished work). Recent advances in MetaQTL analyses and genome wide association mapping approaches (GWAS) can reduce QTL confidence intervals and can help to improve the precision of the number and position of detected QTLs (Courtois et al. [2009], [2013]). These approaches are time consuming, and shortening the timeframe for QTL characterization is clearly a priority for the future.

The most effective strategy for cloning these QTLs appears to be the development of Near-Isogenic Lines (NILs) by marker-assisted selection. These NILs are useful material for fine mapping and QTL cloning but also for breeding and for detailed physiological studies. The NIL strategy combines breeding, exploitation of genetic diversity and positional cloning (Motte et al. [2014]). The work around root architecture and phosphate uptake also illustrates the importance of identifying cultivars that have special behavior (e.g. highly efficient P uptake despite a poor root system) to include rare alleles that will be valuable for breeding. Rice CSSL (Chromosome Segment Substitution Lines) populations, are valuable/natural starting points for QTL mapping and subsequent QTL cloning. Alternatively, developing new populations, especially NILs targeting QTLs of large effect is still a necessary step for its positional cloning.

The rice root system displays a complex structure composed of several tissues and different root types (Rebouillat et al. [2009]). The function of an increasing range of genes involved in rice root development has been deciphered these last 5 years (reviewed in Coudert et al. [2010]; Orman-Ligeza et al. [2013]). Root stress responses are clearly influenced by their cell identity and coordination of cell-specific responses that are currently poorly understood. All of the root tissues, including root cap (Wang et al. [2014]), could develop anatomical and molecular adaptations related to their diverse roles in root growth and function under normal and stress conditions. Exploring root stress responses and adaptations at a cell or tissue specific level by characterization of gene networks involved in these processes is an expanding research field (Gifford et al. [2008]). The use of gene regulatory network inference tools to identify key regulators of root adaptive responses need also to be used in rice. Such an approach should yield candidate master regulators of root responses to stress and is complementary to genetic approaches. The identification of common cis-acting regulatory elements can aid to gather co-expressed genes under root development and stress responses (Pegoraro et al. [2013]).

The validation of QTLs and reverse genetics often involves use of *A. thaliana* data. There is a need to improve and speed up annotation transfer between *Arabidopsis* and rice. Identification of orthologs is probably the most straightforward way. Expression data or synteny can be also used to find the right ortholog(s) between species. Databases of orthologs already exist (Goodstein et al. [2012]; Rouard et al. [2011]) as well as new tools for ortholog prediction (Ostlund et al. [2010]; Schreiber and Sonnhammer, [2013]) but the challenge is to build a simple and functional platform for biologists and breeders.

Functional validation of individual genes associated with root development through knockout or gain-of-function mutants still holds merit. Since the multiple genes of roots are found to have spatial and temporal differences in their expression and function, the search for the trait differences, genes and their function need to be expanded with large set of mutant resources (Wei et al. [2013] for a review). This includes the development of large saturation mutagenized populations, virtually exploring any amino acid variation along the encoded proteins. New gene targeting technologies, mainly TALEN and CRISPR are revolutionizing functional analysis of genes and should be critical to accelerate the characterization of genes of agronomic interest (Gaj et al. [2013]; Miao et al. [2013]). The establishment of an International repository containing CRISPR directed against all rice genes to generate knock-out mutants would be a helpful resource for speeding up gene discovery and validation. Another possibility would be to produce CRISPR in pairs targeting 5’ and 3’ end of each rice gene. This resource would be useful for allele replacement in any rice gene and to validate any QTL effect by introducing only the causative mutation in other genetic backgrounds. Finally, the CRISPR technology allows targeting multiple genes at once in a single transformation event and so opens a simple way to test QTL/gene interactions.

### Establish high throughput phenotyping platforms with relevance to field conditions

GWAS is promoted by the recent progresses in re-sequencing rice genetic resources (CAAS BGI IRRI, [2014]) and reverse and direct genetics approaches are facilitated by the creation of large and diverse mutant collections (Wei et al. [2013]). Dedicated high-throughput phenotyping methods coupled with automated data analysis systems are necessary to fully exploit these genetic resources to identify the genetic determinants of root (adaptive) architecture.

The choice of a phenotyping method always results from a compromise between relevance, precision, throughput, automation and cost to address a specific scientific question. Some key root QTLs have been discovered indirectly without the necessity to screen roots. This is the case of PUP1 that was discovered merely on the basis of the capacity of plants to maintain yield when grown in phosphorus-depleted soils. It is only *a posteriori* that *PSTOL1*, the isolated gene underlying *PUP1* QTL, proved to control an adaptive development response of the root system in low-phosphate soil condition promoting root branching (Gamuyao et al. [2012]). Other important root genetic determinants governing rice adaptation to soil conditions could be found by this indirect way, but it will be probably limited. Most studies performed in the last decades were based on direct observation of the root system *per se*. The use of hydroponics-, soil pots- or soil columns-based systems and the collection of traits such as maximum root length, root thickness, root number, mass of roots at different depths, or root to shoot ratio allowed the identification of several QTLs (Courtois et al. [2009]). Some of these traits have already been integrated into breeding programs, e.g. to increase water deficit tolerance by increasing deep rooting (Shen et al. [2007]). Another soil-based and indirect root screening system uses introduction of herbicide at a deep soil layer (Khalfaoui and Havard, [1993]). In this case the plant starts to die when its root system reaches the herbicide layer. This reflects the rapidity with which the root develops in depth which is a combination of root gravity perception (or root angle) and growth rate (Al-Shugeairy et al. [2014]). Another system, called *shovelomics* uses the partial digging up of the root system under field conditions, through a rapid (8 minutes per plant) phenotyping of some parameters such as root biomass in top soil and the total number of crown roots (Trachsel et al. [2011]). This method gives partial data and does not estimate root depth for example. All these soil-based system have proven their usefulness to discover some major root genes and QTLs. Nevertheless, these systems are destructive and limit fine and dynamic study of root development.

A new alternative experimental set up allows the *in situ* capture of the whole root system in soil pots or columns using X-ray computed tomography (Tracy et al. [2010]; Bao et al. [2014]). This is a non-destructive process, and plants can be imaged at multiple stages of their lifecycle. Though time needed for image acquisition and segmentation currently limits its use to a restricted set of genotypes, it is very promising in term of discovery of genes involved in root developmental adaptive response and deeper characterization of varieties identified through other screens. Other systems focus on early developmental stage and use transparent media that are considered to mimic soil. For example the Rhizoscope, a two-dimensional hydroponic-based system made of plexiglass sandwiches filled with glass beads that mimic soil resistance, allows root system growth up to 30 days after sowing. Root growth can be examined and fine and quantitative parameters can be collected after glass beads removal, root imaging and tissue harvesting. This system has been used to phenotype the roots of a *japonica* rice core collection (Courtois et al. [2013]). *In vitro* agar-medium 3-D based systems have also been developed (Iyer-Pascuzzi et al. [2010]; Clark et al. [2011]). Other systems are restricted to very early developmental stages such as the rolled paper systems where the medium is supplied by capillarity in the support paper and that give access to a bi-dimensional view of the early root system still anchored on the paper after opening (Woll et al. [2005]). These systems allows the following of the dynamics of early root development in a high throughput manner and will be probably very useful to screen the root architecture of rice core or mutant collections and to study the adaptive response of the root system to different nutrients in soil.

Understanding the detailed components of rice root growth, in terms of cell differentiation, anatomy, ion fluxes, and hydraulic conductance requires phenotyping systems that are operational at a finer scale in addition to those characterizing whole root-system architecture. Hydraulic properties of segments of specific root zones were investigated in several cultivars of rice (Ranathunge et al. [2003]). This approach allows a fine description of the water transport capacity along the root, and, in combination with drugs inhibiting the aquaporin intrinsic activity, allows one to decipher the respective contributions of apoplast and cell-to-cell paths (Ranathunge et al. [2004]). The diversity of rice root anatomy is also starting to be systematically explored by image analysis of histological sections. This is promising because the radial tissue differentiation is involved in the capacity of rice to better stand toxicity of minerals such as aluminum and water deficit (Huang et al. [2009]; Henry et al. [2012]). Both tissue section preparation and image analysis can be automated in the future (Périn C., Henry A. pers com). One potential method of investigating 3D cellular structures is the technique of laser ablation tomography that has recently been developed for maize roots (http://plantscience.psu.edu/research/labs/roots/sites/laserfacility). In this technology the surface of a root is alternatively ablated with a pulsed laser and photographed with a dSLR camera. This has now been used in a variety of plant species and is likely to provide a powerful tool for rice research. Four dimensional-reconstruction of a growing rice root tip with cell lineage tracking has also proven feasible (Fernandez et al. [2010]). Recent *Arabidopsis* work has demonstrated that this level of phenotyping allows the GWAS identification of novel important genes involved in root development (Meijon et al. [2014]).

Open and high-throughput root phenotyping platforms that can address root growth in realistic conditions need to be developed in the future. Another important bottleneck is the extraction of meaningful quantitative data from the large amount of images generated by high throughput phenotyping platforms. Several image analysis software products have been developed to assist the root phenotyping platforms (French et al. [2009]; Galkovskyi et al. [2012]; Burton et al. [2012], Clark et al. [2013]; Pound et al. [2013]; Topp et al. [2013]) but so far the image analysis still requires a time-consuming, expert curation step. Although the choice of a phenotyping method is always a compromise between the available means and the scientific question, some reference protocols and parameters as well as priority issue questions should be defined and adopted by the rice community as it has been done for other models (De Smet et al. [2012]). This would allow the rice scientific community to exploit common genetic resources (sequenced genotypes, highly recombinant populations, mutant collections) and to share data such as raw images that could be exploited for different phenotypic traits by different groups. This could apply for early developmental stage with simple standardized culture systems to investigate constitutive and adaptive root development and also for root anatomy. A common repository for these phenotyping data would also pave the way for analyses of genotype x environment interactions, meta-analyses and comparison between different experiments.

### Delineate root ideotypes adapted to diverse target soil environments

For future crops, it is expected that multiple root characteristics should be improved, including those that confer improved water use efficiency and nutrient acquisition (Pennisi [2010]). In the case of rice, one strategy has been to identify genes controlling such interesting root traits, which relies on the direct identification of genetic regions conferring tolerance to a given stress using a genetic screen, GWAS, or QTL analyses (e.g. *PUP1*; Gamuyao et al. [2012]). An alternative is to try to define *a priori* ideotypes of more efficient root systems from our knowledge of soil sciences and plant physiology. Root ideotypes that have been suggested to improve rice performance under drought include increased root growth at depth (Nicou et al. [1970]; O’Toole and Chang [1978]) and coarse axial roots to increase the ability to penetrate hardpans (Hasegawa et al. [1985]). The hypothesis that deep-rooted rice would be more resistant to drought than shallow rooted ones prompted the identification of a gene (*DRO1*) controlling nodal root growth angle in rice, and NIL having the functional allele of *DRO1* showed improved yield under upland drought conditions (Uga et al. [2013]). Similarly, deep rooting has been hypothesized as a trait that could allow for exploitation of soil nutrients at deeper soil layers thus improving mineral uptake and potentially increase yields (Obara et al. [2010]). NILs having the Kasalath allele at QTL qRL6.1 had longer seminal roots than those with the Koshihikari allele under different nitrogen fertilizer concentrations with NH4+ as the source of nitrogen. These examples demonstrate the potential value of ideotype-based approaches. However, as our knowledge of yield determination and response to stress increases, the complexity of the mechanisms involved in rice root response to stress and the diversity of environmental conditions in rice agro-ecosystems make the definition of ideotypes more difficult. Both the approaches of root ideotype-based screening and selection for grain yield are not mutually exclusive and should be conducted in parallel. They would in fact sustain each other; ideotype-based approaches may improve stress response and allow the testing of the ideotypes, and yield-based screens will provide better knowledge of the physiology of stress tolerance that will help refine the ideotypes. Such refined ideotypes should lead our formation of strategies to design new genetic screens based on a better knowledge of the integrated stress responses (physiology, development and plant-microbe interactions) and might help identify interesting alleles that might not be identified otherwise. For example, an experimental approach based on better knowledge of the physiology of water transport/acquisition has the potential to provide more detailed insights to mechanisms behind drought tolerance than a screen based on grain yield alone. Two major factors need to be addressed in order to optimize the benefit of such an ideotype-based approach: the understanding of environmental conditions and the integration of the complex factors behind stress response.

To optimize the outputs of root biology for crop improvement, it is first important to better define the environmental stresses that are faced by rice culture worldwide. This points to the need for better characterization of the actual (on-farm) target environments in which crops are grown (including soil physical properties, as in the approaches reported by Inthavong et al. [2011] or Haefele et al. [2014]), and the need for good estimates of future scenarios for environmental conditions faced by rice growers. This will help define the major limiting factors that can be addressed through root ideotype breeding, and to target the most important varieties specific to target each type of environmental conditions to maintain food production in the future (i.e., upland and rainfed lowland drought stress, or fluctuating soil moisture conditions).

Plant responses to environmental stresses are complex and involve multiple levels of integration. Root architecture has been the focus of most proposed ideotypes in the past but anatomical and physiological traits are likely to play equally key roles. Some research on water transport ideotypes has been conducted through studies on the effect of xylem vessel diameter (Yambao et al. [1992]; Henry et al. [2012]) but there is not a consensus in the literature as to whether larger or smaller xylem vessel diameters are ideal. Establishing, a definite answer to these issues will be important, although it should be borne in mind that different xylem diameters/configurations may be optimal for different environments. Given the predicted increasing fluctuations in rice-growing climates, genotypes exhibiting a high degree of root growth plasticity will likely increase in importance (Kano et al. [2011]). Phenotypic effects of beneficial microbes (fungi and bacteria) on plant root system include primary and lateral root length, number and positioning of lateral roots, and overall root growth (Dodd and Ruiz-Lozano [2012], Sukumar et al. [2013]). For instance, it has been shown in rice that the arbuscular mycorrhizal (AM) fungus, *Rhizophagus irregularis*, stimulates the formation of fine and large lateral roots (Gutjahr et al. [2009]). On the other hand, AM fungi form hyphal networks which operate as functional extensions of the plant root system (Smith & Read [2008]). Hence, it appears that beneficial microbes can be considered as an integral part of the root system, from which they significantly contribute to nutrient acquisition and host tolerance to occasional or prolonged abiotic and biotic stress. Our perspective is that, in addition to root architecture, future work on ideotypes should integrate functional parameters (e.g. water and ion uptake per length of root and the respective contributions of apoplast and cell-to-cell paths), plasticity, and plant/soil microbe interactions which can alter host root development and functioning.

However, as our understanding increases, the concept of root ideotype is less clearly defined. It is therefore important to exploit the power of modeling to integrate these different factors and be able to make predictions about the traits that may be beneficial. Modeling forces us to examine many parameters related to a specific process and allows us to test the sensitivity of the model to each of these. This can inform about potential rate-limiting processes that govern complex traits such as water or nutrient acquisition and guide the subsequent identification of associated QTLs. Several structural functional root models have been published (e.g. SimRoot, Lynch et al. [1997] and R-SWMS, Dunbabin et al. [2013]) as well as other soil models (Reviewed in Hill et al. [2013]). Root/soil interaction models will be important in simulating the dynamic response and efficiency of different rice ideotypes in different soil conditions. One considerable advantage of such models is that they allow us to investigate the cost-benefit relationship between various traits in a wide variety of environments. For example, producing longer roots can protect against drought but it comes at a cost of carbon that could be used elsewhere, and deep roots may be less efficient in foraging for nitrogen. Structural-functional plant models based on an economy of resources allow us to identify potential combinations of traits that can increase plant growth under a wide variety of environmental conditions, including water or nutrient poor conditions. These predictions could drive breeders or genetic engineers towards producing plants with these combinations of traits, so that the models can be tested experimentally. Again, characterization of the environment will be critical for this analysis, and having a geographically distributed network of rice root researchers with knowledge of different soils is an enormous asset. Future models need to take into account the soil structure as well as nutrient/water availability, and incorporate both root architecture and anatomy within the model. These tools will allow the search for more difficult to study root traits that are linked to adaptability, rather than fixed characters for a given environment. This effort should be community-based and where possible should use open access platforms (such as OpenAlea; Pradal et al. [2008]) to guarantee adoption and the continuity of curation of the developed models.

In summary, we think that in addition to the approach of screening for stress tolerance among diverse rice genotypes (including wild relatives), there are many benefits to be gained from developing an approach to root biology based on the integration of our knowledge of the physiology of plant response to stress to define ideotypes that can form the basis of new more specific genetic screens. For instance, our perspective is that future work on ideotypes should integrate functional parameters (i.e. water and nutrient uptake per length of root,). Future research is necessary to conceptualize new integrative root ideotypes that will enable the definition of new experimental screening approaches to contribute to rice improvement strategies. A more complex, but necessary step would be the coupling of the roots development modelling approaches and tools with the ones of the whole plant modeling in order to reason the ideotype at the whole plant level.

### Breed for improved root systems of future rice varieties

Several hundred QTLs for root traits (mainly structural and a few functional) have been identified and compiled for the identification of meta-QTLs (Courtois et al. [2009]; Khowaja et al. [2009]). Association mapping, which improves further the resolution in QTL position, has already been initiated for rice root traits on broad-based panels (Famoso et al. [2011]; Courtois et al. [2013]; Wissuva et al. unpublished work) and a core collection of local varieties from Vietnam (Gantet et al. unpublished work). One of these QTLs, *DRO1,* was recently cloned and its function characterized (Uga et al. [2013]). More recently, the characterization of QTLs with large effect involved in phosphorus uptake (Gamuyao et al. [2012]) and in yield under drought (Kumar et al. [2013]; Swamy et al. [2013]) revealed direct relationship with or contribution from root traits. Efforts for cloning a number of other root QTLs are ongoing at NIAS (QTLs for stele size and surface rooting), JIRCAS (QTLs for P uptake), Nagoya University (QTL for root growth in response to soil moisture fluctuations and root growth plasticity) IRRI (QTLs for yield under drought), CIRAD (QTLs for root length) and Tamil Nadu University (QTLs for root penetration ability). Thus a large number root QTLs or genes promising for improving rice performances in water and nutrient uptake are now available for breeders.

Efforts in marker assisted introgression of root QTLs from landraces into elite material go back to 2001 (Shen et al. 2001) with mixed effects on yield in target environment in some cases (Steele et al. 2007). positive effects in some other cases (Gamuyao et al. [2012]; Uga et al. [2013]; Steele et al. [2013]). An important next step for practical use of the large number of root QTLs within breeding programs would be their characterization for QTL x environment and QTL x genetic background interactions, using grain yield as the selection criterion. Indeed, root-related QTLs/genes may interact with the genetic background into which they are introgressed and with the environment to which they are targeted. Thus, it will be necessary to assess the effect of individual or combination of QTLs in the largest possible number of relevant environments and genetic backgrounds, the latest taking into account the structuration of *O. sativa* into four majors groups (*indica, japonica, au* s and *aromatic*). The systematic implementation of such an approach (all combinations of genetic backgrounds and environments) would necessitate a too large set of material to be developed. Simulation modeling could be useful to help discard non-functional trait/QTL combinations at early stages in the development of improved rice genotypes. Subsequently, the results of the yield evaluation together with existing QTL data could be consolidated to develop a breeder’s chip carrying markers associated with the main QTLs and candidate genes.

We expect that as more QTLs/target genes are identified and as genetic and genomic approaches converge, a radical change in methodology to rapidly deliver new rice varieties will be necessary. Root architecture is one trait among others for breeders, and it will be necessary to concurrently manipulate or introgress in addition to genes related to root architecture, those for disease resistance, multiple abiotic stress tolerance, grain quality, etc. To deal with such complexity, an emerging solution is genomic selection using a multi-trait index (Meuwissen et al. [2001]). The methodology is being adapted to plants and shows interesting promises (Jannink et al. [2010]; Lorenz et al. [2011]). However the application of genomic selection will require making parallel progress in high throughput phenotyping to calibrate predicting models, in extraction of DNA of the right quality for a high number of plants in a short time, and decreasing even more the cost of genotyping.

To take full advantage of the potential of genomic selection, the process of recombination of multiple parents will also have to be rationalized and accelerated. The best solution to accumulate favorable alleles at multiple genes in a unique background is probably not to try to do it in one round. Since breeding is a numbers game, this would require populations that are too large. Theory demonstrated that a two or more step procedure is much more efficient although slightly slower (Kervella et al. [1993]). Recurrent selection with a male sterility gene to facilitate intercrossing will be a way to go (Grenier et al. unpublished work). As part of the favorable alleles may come from exotic material, the issue of introgression of the genes into an advanced background without disturbing the whole genetic balance has to be solved. This may mean using bridges between wild species or landraces and the elite gene pools. These approaches should help breeders in taking further advantage of the genetic diversity of root traits for improving rice for future harvests.

## Conclusion

### The roots of future rice harvests

In summary, we identified the following priority targets for rice root research during the workshop:

Have a better definition of the environmental stresses that are faced by rice crop worldwide and a better characterization of the actual (on-farm) target environments. For this and to challenge ideotype models, we must mobilize a geographically distributed network of rice root researchers with knowledge of different soils.

Create new populations and new NILs for root QTL cloning

Explore genes and alleles in wild relatives and landraces without neglecting the modern gene pools that can probably also provide interesting features that will be easier to transfer to cultivated rice.

Challenge the effect of the QTLs in several genetic backgrounds taking into account the strong species structuration, across ecosystems and then within each ecosystem. QTL expression has to be analyzed in the largest possible number of relevant situations.

Gain detailed knowledge on the roles that genes corresponding to QTLs play in root and plant physiology (i.e. mineral and water nutrition). Organize/stimulate the exchange of information between research groups aiming at identifying QTLs/having identified QTLs and groups likely to be interested by the investigation of the roles that the corresponding QTL play in root and plant physiology.

Develop tools for ortholog prediction between rice, *Arabidopsis* and crops to accelerate candidate validation and information transfer

Integrate genome editing and allele replacement tools in root QTL validation

Study how roots regulate stress responses at cell/tissue levels through cell specific *omics* approaches and cell-specific gene expression.

Better understand the epigenetic regulation of roots and root development under stressful conditions

Prepare for a capacity of accumulating favorable alleles at multiple genes in a unique background, notably through pyramiding and recurrent selection and establishing intermediary bridges for those residing in exotic germplasm.

Accelerate the development of dedicated high throughput phenotyping systems coupled with automated data analysis

Adopt joint reference protocols and parameters as well as priority issue questions allowing a common and shared effort of harnessing of genetic resources. Create a common phenotyping data repository for comparisons and meta-analyses.

Promote the approaches coupling complementary root ideotype-based screening and selection for grain yield, each approach sustaining the other.

Design new genetic screening methods based on a better knowledge of the integrated stress responses. Integrate functional parameters (e.g.. water uptake per length of root), plasticity, and plant/soil microbe interactions.

Intensify the efforts towards dynamic root/soil interaction modeling to integrate these different factors and be able to make predictions about the traits that may be beneficial under a variety of environmental conditions.

The root system being less accessible and more complex than other agronomical traits, achieving the ambitious goal of breeding the roots of the future requires a coordinated effort and joint resources. The GRiSP workshop held in Montpellier has confirmed that an expanding community, including groups previously using *Arabidopsis* as a model, is at work to tackle the characterization of the structure and function of rice roots and to integrate root traits for breeding improved varieties. This first workshop had no ambition to gather the whole community and tackle all the complex aspects of rice root research. Participants were conscious that many other groups targeting complementary aspects of root biology and ecology research in rice do exist and can join their efforts to this community. Recent advances have been accomplished in the genetic and molecular control of root development, root system phenotyping, imaging and modeling as well as in genome-wide marker assisted breeding and access to nucleotide variation in genetic resources. Altogether, this makes a unique conjunction for a community to build a promising and exciting future in rice root research. In that aim we call for a better coordination of research and breeding efforts through the creation of a strong comprehensive rice root research network as well as for appropriate support from national and international funding bodies. These efforts are both worthwhile and timely since roots are certainly one of the key traits which will help us to adjust crops to the predicted increasing climate instability and resource scarcity with the objective of feeding 9 billion people by year 2050.
